# Amalgam Crown

**Published:** 1900-10-15

**Authors:** H. Barnes


### AMALGAM CROWN.

A sectional plaster model is taken of the crown of a natural molar tooth. Different models will give a selection of crowns, and they can be made in the laboratory by the assistant, and kept on hand for use at any time.

Quick-setting amalgam is then burnished to the sides of the model, leaving the center hollow and a hole through the center of the crown. When the amalgam has set it is polished, and you have a hollow amalgam crown which may be set upon a badly decayed root with quick-setting amalgam.

The root is prepared as for any large filling, with pins extending into the coronal portion; a crown is selected of the correct size and shape; a narrow band of phosphor bronze is placed about the cervical portion of the root, extending up to hold the crown in position. Prepare amalgam rather soft and pack into the root portion; the crown is then forced into position, being held in place by the band. The hole in the top of the crown permits of manipulation from above with instruments. When on a few minutes, the band may be removed and the excess of amalgam about the cervical portion removed in the usual manner. Those who have seen the crown here are quite well pleased with it.—*H. Barnes, in International Journal.*

